# Supplementary material for: Stockpiled personal protective equipment and knowledge of pandemic plans as predictors of perceived pandemic preparedness among German general practitioners
Source: PLoS One. 2021 Aug 12;16(8):e0255986. doi: 10.1371/journal.pone.0255986 (PMC8360569; doi:10.1371/journal.pone.0255986)
Supplement: S1 Table — (DOCX) [file pone.0255986.s001.docx]

S1 Table. Questionnaire (German and English translation)

Questionnaire (German, original version)

| Wie gut haben Sie sich Anfang März mit Ihrer Praxis auf eine Epidemie vorbereitet gefühlt? [pand_pre] | sehr schlecht [1]  schlecht [2]  teils teils [3]  gut [4]  sehr gut [5] |
| --- | --- |
| Wie stellte sich Anfang März Ihr Bestand an Schutz- und Hygienematerialien dar? | |
| FFP-2 und FFP-3 Masken [PPE_FFPmasks] | für unsere Praxis nicht relevant [0]; völlig unzureichend [1]; unzureichend [2]; ausreichend [3]; völlig ausreichend [4] |
| Mund-Nasen-Schutz [PPE_medmasks] | für unsere Praxis nicht relevant [0]; völlig unzureichend [1]; unzureichend [2]; ausreichend [3]; völlig ausreichend [4] |
| Einmalhandschuhe [PPE_gloves] | für unsere Praxis nicht relevant [0]; völlig unzureichend [1]; unzureichend [2]; ausreichend [3]; völlig ausreichend [4] |
| Hände- und Flächendesinfektionsmittel [PPE_dis] | für unsere Praxis nicht relevant [0]; völlig unzureichend [1]; unzureichend [2]; ausreichend [3]; völlig ausreichend [4] |
| Schutzbrillen [PPE_glasses] | für unsere Praxis nicht relevant [0]; völlig unzureichend [1]; unzureichend [2]; ausreichend [3]; völlig ausreichend [4] |
| Schutzanzüge [PPE_suit] | für unsere Praxis nicht relevant [0]; völlig unzureichend [1]; unzureichend [2]; ausreichend [3]; völlig ausreichend [4] |
| Gesichtsschutzschilder [PPE_shields] | für unsere Praxis nicht relevant [0]; völlig unzureichend [1]; unzureichend [2]; ausreichend [3]; völlig ausreichend [4] |
| Waren Ihnen vor dem Ausbruch der Corona-Pandemie Anfang März Pandemie- oder Epidemiepläne bekannt? [plan_knowledge] | Ja [1]  Nein [0] |
| Haben Ihnen diese Pandemie- oder Epidemiepläne für die Bewältigung der Corona-Pandemie geholfen? [plan_helpful] | Ja [1]  Nein [0] |
| Wie alt sind Sie? [age] | unter 30 Jahre [1]  31 bis 40 Jahre [2]  41 bis 50 Jahre [3]  51 bis 60 Jahre [4]  über 60 Jahre [5] |
| Welchem Geschlecht fühlen Sie sich zugehörig? [gender] | männlich [1]  weiblich [2]  divers [3] |
| Sind Sie in der Praxis selbstständig oder angestellt tätig? [empl] | selbstständig [1]  angestellt [2] |

Questionnaire (English, translation)

| How prepared did you feel at your practice for a pandemic in early March? [pand_pre] | very bad [1]  bad [2]  moderate [3]  good [4]  very good [5] |
| --- | --- |
| As of early March, what was your inventory of the following protective and hygienic materials? | |
| FFP-2/3 masks [PPE_FFPmasks] | not relevant [0]; completely insufficient [1]; insufficient [2]; sufficient [3]; completely sufficient [4] |
| medical masks [PPE_medmasks] | not relevant [0]; completely insufficient [1]; insufficient [2]; sufficient [3]; completely sufficient [4] |
| surgical gloves [PPE_gloves] | not relevant [0]; completely insufficient [1]; insufficient [2]; sufficient [3]; completely sufficient [4] |
| Hand and surface disinfectants [PPE_dis] | not relevant [0]; completely insufficient [1]; insufficient [2]; sufficient [3]; completely sufficient [4] |
| safety glasses [PPE_glasses] | not relevant [0]; completely insufficient [1]; insufficient [2]; sufficient [3]; completely sufficient [4] |
| protective suit [PPE_suit] | not relevant [0]; completely insufficient [1]; insufficient [2]; sufficient [3]; completely sufficient [4] |
| face shields [PPE_shields] | not relevant [0]; completely insufficient [1]; insufficient [2]; sufficient [3]; completely sufficient [4] |
| Were you aware of any pandemic or epidemic plans prior to the Corona pandemic outbreak in early March? [plan_knowledge] | Yes [1]  No [0] |
| Did these pandemic or epidemic plans help you manage the Corona pandemic? [plan_helpful] | Yes [1]  No [0] |
| How old are you? [age] | 30 years and younger [1]  31 to 40 years [2]  41 to 50 years [3]  51 to 60 years [4]  older than 60 years [5] |
| Which gender do you feel you belong to? [gender] | male [1]  female [2]  none-binary [3] |
| Are you self-employed or employed in the practice? [empl] | self-employed [1]  employed [2] |
