# Supplementary material for: Stockpiled personal protective equipment and knowledge of pandemic plans as predictors of perceived pandemic preparedness among German general practitioners
Source: PLoS One. 2021 Aug 12;16(8):e0255986. doi: 10.1371/journal.pone.0255986 (PMC8360569; doi:10.1371/journal.pone.0255986)
Supplement: S2 Table — (DOCX) [file pone.0255986.s002.docx]

S2 Table. Key sociodemographic characteristics

| Variables | n (%) |
| --- | --- |
| Age (n = 507)  *30 years and younger [1]*  *31 to 40 years [2]*  *41 to 50 years [3]*  *51 to 60 years [4]*  *older than 60 years [5]*  *missings* | -  56 (11.05)  129 (25.44)  207 (40.83)  115 (22.68)  1 |
| Gender (n = 508)  *male [1]*  *female [2]*  *none-binary [3]*  *missings* | 265 (52.16)  242 (47.64)  1 (.00)  - |
| Employment status (n = 505)  *self-employed [1]*  *employed [2]*  *missings* | 458 (90.69)  47 (9.31)  3 |
| Federal states (n = 501)  *Baden-Württemberg [1]*  *Bavaria [2]*  *Berlin [3]*  *Brandenburg [4]*  *Bremen [5]*  *Hamburg [6]*  *Hesse [7]*  *Mecklenburg-Western Pomerania [8]*  *Lower Saxony [9]*  *North Rhine-Westphalia [10*  *Rhineland-Palatinate [11]*  *Saarland [12]*  *Saxony [13]*  *Saxony-Anhalt [14]*  *Schleswig-Holstein [15]*  *Thuringia [16]*  *missings* | 71 (14.17)  93 (18.56)  23 (4.59)  5 (1.00)  3 (.60)  8 (1.60)  64 (12.77)  13 (2.59)  45 (8.98)  95 (18.97)  12 (2.40  11 (2.20)  26 (5.19)  11 (2.20)  16 (3.19)  5 (1.00)  7 |
| Practice type (n = 506)  *Single practice [1]*  *Group practice [2]*  *missings* | 249 (49.21)  257 (50.79)  2 |
| Practice location (n = 503)  *<5.000 inhabitants [1]*  *>5.000-20.000 inhabitants [2]*  *>20.000-100.000 inhabitants [3]*  *>100.000 inhabitants [4]*  *missings* | 103 (20.48)  123 (24.45)  130 (25.84)  147 (29.22)  5 |
