# Supplementary material for: Stockpiled personal protective equipment and knowledge of pandemic plans as predictors of perceived pandemic preparedness among German general practitioners
Source: PLoS One. 2021 Aug 12;16(8):e0255986. doi: 10.1371/journal.pone.0255986 (PMC8360569; doi:10.1371/journal.pone.0255986)
Supplement: Attachment — Submitted filename: Response to Reviewers.docx [file pone.0255986.s003.docx]

Response to Reviewers

**Manuscript ID:** **PONE-D-21-13972**

**Title: Stockpiled personal protective equipment and knowledge of pandemic plans as predictors of perceived pandemic preparedness among German general practitioners**

Dear Jianguo Wang, (Academic Editor, PLoS ONE)

Thank you for your response to the submission of our manuscript. We are very grateful for the opportunity to re-submit it to PLoS ONE.

Below are the comments from the reviewers and how we responded to their comments.

Thank you for this opportunity to address their concerns.

Sincerely,

Arno Stöcker

| Comment | Response |
| --- | --- |
| Editorial staff comments | |
| Please review your reference list to ensure that it is complete and correct. If you have cited papers that have been retracted, please include the rationale for doing so in the manuscript text, or remove these references and replace them with relevant current references. Any changes to the reference list should be mentioned in the rebuttal letter that accompanies your revised manuscript. If you need to cite a retracted article, indicate the article’s retracted status in the References list and also include a citation and full reference for the retraction notice. | References have been reviewed. |
| 1. Please ensure that your manuscript meets PLOS ONE's style requirements, including those for file naming. The PLOS ONE style templates can be found at:  https://journals.plos.org/plosone/s/file?id=wjVg/PLOSOne_formatting_sample_main_body.pdf  and  https://journals.plos.org/plosone/s/file?id=ba62/PLOSOne_formatting_sample_title_authors_affiliations.pdf | Style requirements have been reviewed. The affiliation and corresponding author segment have been updated (lines 18-22). |
| 2. Please include additional information regarding the survey or questionnaire used in the study and ensure that you have provided sufficient details that others could replicate the analyses. For instance, if you developed a questionnaire as part of this study and it is not under a copyright more restrictive than CC-BY, please include a copy, in both the original language and English, as Supporting Information.  Furthermore, in your Methods section, please provide a justification for the sample size used in your study, including any relevant power calculations (if applicable). | Thank you for your important comment on the reproducibility of our study. The essential questions are already available in the text (Rev. manuscript, lines 119-125). For a better overview, we have compiled the questions in German and English translation into a separate document and attached it in the supplement (S1 Table).  Since this is an exploratory study, no power calculation was performed. We have made the following addition in the methods section (lines 103-104):  “The study population was selected to capture outpatient care during the Corona pandemic from the perspective of different medical disciplines.” |
| 3. Please provide additional details regarding participant consent. In the ethics statement in the Methods and online submission information, please ensure that you have specified (1) whether consent was suitably informed and (2) what type you obtained (for instance, written or verbal). If your study included minors under age 18, state whether you obtained consent from parents or guardians. If the need for consent was waived by the ethics committee, please include this information. | Thank you for your comment on the more detailed description of the study participation. Since our survey was directed at physicians, no persons under 18 years of age were contacted and asked to participate. We have updated and added the following section to the methods section (lines 109-116):  “The survey *(including invitation letter, study and privacy information and questionnaire)* was approved by the Ethics Committee of the University of Cologne (20-1169_1). The online survey was conducted anonymously, without directly collecting personal identifying information, so that only implicit consent had to be obtained in accordance with the ethics vote of the Ethics Committee of the University Hospital of Cologne. The terms and conditions of the study had to be agreed to in order to participate in the study. Participation in the survey could be terminated at any time. The possibility to pause the survey and continue it later was technically possible. Participation was voluntary for all participants. No expense allowance or payment was paid for participation.” |
| 4. We note that you have stated that you will provide repository information for your data at acceptance. Should your manuscript be accepted for publication, we will hold it until you provide the relevant accession numbers or DOIs necessary to access your data. If you wish to make changes to your Data Availability statement, please describe these changes in your cover letter and we will update your Data Availability statement to reflect the information you provide. | We have uploaded a Data Review URL in the editorial system. |
| 5. Please include a copy of Table 5 which you refer to in your text on page 15. | Thank you for your attentive comment. The table on page 15 was incorrectly marked as Table 4. We have checked all table numbering again. |
| Reviewer 1 | |
| Thank you for the chance to review this manuscript. I thought that this was generally a well-written manuscript focusing on the association between sufficiency of various PPE stocks and perceived pandemic preparedness among German GPs.  The authors found that PPE stocks in general seem to be significant associated with PP scores, and that facial protection in particular was a prominent piece of PPE. Both of these findings seem logical to me given the understanding of the COVID-19 situation and known transmission mechanics at the time that the study was conducted.  I also felt that the authors used appropriate regression analyses that were well detailed in the Results section, and the Discussion was able to present an interpretation without overclaiming the validity of the findings.  I just have two minor comments for the authors to improve their manuscript for potential publication: | Thank you for your review and appreciative comments. |
| 1. I understand from your Methods section that 6,500 GPs were invited to participate, of which 535 GPs responded. This is a very low response rate of approximately 8.2%.  How might this low response rate have affected the generalisability of your study's findings? Given that your survey was online and anonymous, would it have been possible for certain selection biases (e.g., location, socio-economic status) to affect the findings? Is there a possibility of data errors (e.g., same GP responding to the survey more than once)?  If there is insufficient data (due to anonymity of the respondents) to establish the presence of these biases, it would be good to elaborate on them in the limitations section. | Thank you for your important feedback on the number of participants and the transferability of the results. We have added the following in the limitations (see also response to your next comment) (lines 315-319).  “Because this is an anonymous survey, it cannot be ruled out that participants may have responded to the survey more than once or that non-physicians participated. Though, at the beginning of the survey, it was asked whether the participant works as a physician in the outpatient sector. If this answer was negative, participation in the survey was terminated. Nevertheless, it cannot be ruled out that deliberately false statements were made here.” |
| 2. On line 121, you mention that the study population of GPs was relatively homogeneous as a way of justifying why only age and gender were chosen as potential confounders. It would be ideal for you to demonstrate to the reader that this homogeneity in your study sample is actually true. For example, you could present key sociodemographic characteristics of the sample in a descriptive table. | Thank you for this comment. We were able to identify several known sociodemographic factors that give hope to the tendency that the study population matches the population of German general practitioners in essential aspects. We have adjusted the limitations accordingly (lines 319-338):  “The selected recruitment method does not allow representative conclusions for GPs in Germany. However, if we consider key sociodemographic characteristics of the study participants (S2 Table) and compare them with the basic population of German GPs, it becomes clear that there are indications that the study population represents German GPs reasonably accurately. First of all, participants from all 16 German federal states and city states took part in the study. In view of the statistical data from the German Federal Register of Physicians, it appears that our study population was, on average, somewhat younger than the average German GPs (approximately 53 years compared to 55.4 years). With regard to the gender distribution of the sample, this corresponds to the national average for GPs (52% male, 48% female) provided by the Federal Register of Physicians. About 90 % of the physicians surveyed reported that they are self-employed. This is about 10 percentage points higher than the national average from the German Medical Association. The overrepresentation of self-employed physicians can possibly be explained by the fact that they were contacted via fax. Although the invitation letters were personalized, the faxes may nevertheless have been presented to the practice owner. Also, in the case of practice email addresses, the practice owner may have been the primary contact or may have had access first. It is also possible that self-employed physicians have a higher level of commitment and identification with their own profession, so that a slight selection bias cannot be ruled out. Furthermore, a possible selections bias may also have implications for the reported pandemic preparedness. More job committed individuals may also have higher general preparedness. As a result, this could lead to a slight overestimation of the PP of the analyses in the population." |
| Reviewer 2 | |
| Hand hygiene is a very important part of Personal Protective Equipment - however in the manuscript data on hand hygiene presence (I assume alcohol-based handrub) is presented combined with surface disinfectant - the two should be distinguished from each other since different use and process and this change should be reflected in the result section also | Thank you for this critically in-depth comment. The survey queried the inventory of hand and surface disinfectants together in one item, which is why no more specific statements on the individual disinfectants could be made in the analyses. We have included this point in the limitations (lines 312-314):  “With regard to the provision of disinfectants, the inventory of hand and surface disinfectants was queried together. Accordingly, this survey cannot provide more precise information on the distinction between the two PPE materials.” |
| In results Table 2 - can you clarify if the sanitizer is hand sanitizer? | Thank you for the clarifying note. We have adjusted the labeling in Table 2 to „hand and surface disinfectants“. |
| Can you identify any psychometric testing/ validation/piloting performed of the survey instruments? What about validation of the survey instrument answers? Was there a sample of completed forms that were validated? | Thank you for this important comment. Since this is an exploratory study and no psychometric questions or scales were used, we did not validate the questions. We have included a list of the questions in German and English translation as an appendix (S1 Table). For the PPE score, variance inflation factor and tolerance were specified (Table 2); and Cronbach's alpha indicated a sufficient reliability and internal consistency of the PPE score (0.81 (CI:.78;.83)). We added the following to the methods section on questionnaire design (lines 96-99):  "During questionnaire conception and development, preliminary interviews were conducted with different representatives of the listed group of specialists. The questionnaire was subsequently tested by several physicians from different specialty groups who were not involved in the design." |
