# Supplementary material for: Stockpiled personal protective equipment and knowledge of pandemic plans as predictors of perceived pandemic preparedness among German general practitioners
Source: PLoS One. 2021 Aug 12;16(8):e0255986. doi: 10.1371/journal.pone.0255986 (PMC8360569; doi:10.1371/journal.pone.0255986)
Supplement: Attachment — Submitted filename: Response to Reviewers.docx [file pone.0255986.s004.docx]

Response to Reviewers

**Manuscript ID:** **PONE-D-21-13972**

**Title: Stockpiled personal protective equipment and knowledge of pandemic plans as predictors of perceived pandemic preparedness among German general practitioners**

Dear Jianguo Wang, (Academic Editor, PLoS ONE)

Thank you again for your response to the submission of our manuscript. We are very grateful for the opportunity to re-submit it to PLoS ONE.

Below are the comments from the reviewers and how we responded to their comments.

Thank you for this opportunity to address their concerns.

Sincerely,

Arno Stöcker

| Comment | Response |
| --- | --- |
| Editorial staff comments | |
| No Response |  |
| Reviewer 1 | |
| Limitations (page 21)  - Thank you for elaborating in detail about the limitations of an anonymous online survey, as well as the attempt to relate your sample's sociodemographic distribution to national demographic sources for German GPs. However, I would suggest that you cite the respective sources (e.g., Federal Register of Physicians, German Medical Association). Otherwise it is difficult for the reader to access and compare the relevant demographic characteristics accordingly. | Thank you for this helpful comment for an easier and improved comprehension for readers who are not familiar with the German health care system. We have supplemented the sources accordingly (45-47). |
| Reviewer 2 | |
| No Response |  |
